# A broad comparative genomics approach to understanding the pathogenicity of Complex I mutations

**DOI:** 10.1038/s41598-021-98360-7

**Published:** 2021-10-01

**Authors:** Galya V. Klink, Hannah O’Keefe, Amrita Gogna, Georgii A. Bazykin, Joanna L. Elson

**Affiliations:** 1 Sector of Molecular Evolution, Institute for Information Transmission Problems (Kharkevich Institute) of the Russian Academy of Sciences, Moscow, Russian Federation; 2 Population Health Sciences Institute, Newcastle University, Newcastle upon Tyne, UK; 3 Biosciences Institute, Newcastle University, Newcastle upon Tyne, UK; 4 Center of Life Sciences, Skolkovo Institute of Science and Technology, Skolkovo, Russian Federation; 5 Human Metabolomics, North-West University, Potchefstroom, South Africa

**Keywords:** Evolution, Genetics, Evolutionary biology, Mutation

## Abstract

Disease caused by mutations of mitochondrial DNA (mtDNA) are highly variable in both presentation and penetrance. Over the last 30 years, clinical recognition of this group of diseases has increased. It has been suggested that haplogroup background could influence the penetrance and presentation of disease-causing mutations; however, to date there is only one well-established example of such an effect: the increased penetrance of two Complex I Leber's hereditary optic neuropathy mutations on a haplogroup J background. This paper conducts the most extensive investigation to date into the importance of haplogroup context in the pathogenicity of mtDNA mutations in Complex I. We searched for proven human point mutations across more than 900 metazoans finding human disease-causing mutations and potential masking variants. We found more than a half of human pathogenic variants as compensated pathogenic deviations (CPD) in at least in one animal species from our multiple sequence alignments. Some variants were found in many species, and some were even the most prevalent amino acids across our dataset. Variants were also found in other primates, and in such cases, we looked for non-human amino acids in sites with high probability to interact with the CPD in folded protein. Using this “local interactions” approach allowed us to find potential masking substitutions in other amino acid sites. We suggest that the masking variants might arise in humans, resulting in variability of mutation effect in our species.

## Introduction

Mitochondria are maternally inherited^1^, which means the evolution of mitochondrial DNA (mtDNA) is marked by the emergence of distinct lineages, called haplogroups^2,3^. Another aspect of mitochondrial genetics is that mtDNA accumulates single nucleotide variants (SNVs) at a higher rate than nuclear DNA, about tenfold higher^4^. This increased mutation rate also results in a large number of homoplasies or parallel mutations. These are highly useful for making evolutionary inferences about allele preferences at specific sites, and thus the fitness of alternative amino acids^5,6^.

MtDNA is an ~ 16Kbp circular chromosome encoding 13 proteins, 22 tRNAs and 2 rRNAs. MtDNA copy number is linked to the cell’s energetic demands, ranging from hundreds to thousands of copies per cell^7^. The expected state in cells or tissues is that all mitochondrial sequences are the same, meaning the mtDNA is homoplasmic; however, more than one mtDNA genotype can exist in cells, these genotypes usually differ at a single site, this is a state known as heteroplasmy. Heteroplasmy is exhibited in patients with mitochondrial disorders, where a pathogenic mutation is seen as one of the genotypes, alongside the wild-type. Mutations of mtDNA are an important cause of inherited disease in humans.

Diseases caused by mtDNA exhibit a high degree of clinical heterogeneity, with mitochondrial disorders having been most widely studied in patients having Caucasian-European haplogroups: H, V, U, K, T, J, I, X and W. The Yarham et al. pathogenicity scoring system is widely used in the mitochondrial medical community to link genotype to phenotype, drawing together wet-lab and evolutionary science in the diagnosis of mt-tRNA mutations^8^. This has allowed a large number of proven disease-causing mutations to be documented; however, these mutations might not cause disease in all populations. Research in Black South African populations indicates that different mutations might be important in these populations^9^. The phenotypic presentation of mitochondrial disease-causing mutations is also thought by some to differ between populations^10^.

Non-human species can provide a means of exploring mtDNA sequences to better understand the impact of sequence context on the expression of mtDNA variants linked to disease. That is to say, if a proven point mutation associated with disease in humans is present in non-human animals but they do not have a phenotypic manifestation of disease (so-called compensated pathogenic deviations, or CPD), then exploring the surrounding sequence context can give us insight into the importance of haplogroup context in the presentation and manifestation of mtDNA disease. Previous research has shown that non-human species do harbor disease-associated point mutations without the presence of disease. Magalhaes J. found 46 human 'disease-associated' mutations across the consensus mitochondrial genomes of 12 primates^11^. Similarly, Kern and Kondrashov^12^ used single sequences from 106 species, identifying 52 ‘pathogenic mutations’ across the mt-tRNAs; however^12^, the existence of an accepted methodology to link genotype to phenotype had not yet become available at the time of publication of either of these studies. Thus, these prior studies looked at purported disease-causing mutations with weak evidence to support a link between the mtDNA variants and disease in humans, many of their examples were later demonstrated to be population variants.

Queen et al.^13^ performed a study of 33 non-human species using at least 30 high-quality sequences from each species. The paper of Queen et al. investigated the mitochondrial point mutation, m.3243A > G in detail, which has been identified as the most prevalent point mutation in humans^13^. Thus, the linkage of genotype to phenotype was not controversial^14,15^. The m.3243A > G mutation was seen in 57/391 of *Canis lupus familiaris* (dog) sequences retrieved from Genbank. Exploration of the mt-tRNA-LEU(UUR) gene in *Canis lupus familiaris* revealed two variants, which change a G:U Wobble base pair and a mismatched base pair to a Watson–Crick pairing within the D-stem of *Canis lupus familiaris*. Thus, these variants resulted in changes to the secondary structure, which is considered to be a possible mechanism for masking the pathogenic effects of m.3243A > G by the introduction of greater stability to the D-stem^13^. The observation of the importance of sequence context in this group of genes, the mt-tRNAs, was confirmed by a subsequent study looking at the remaining 21 mt-tRNAs encoded for by mtDNA where many more such examples of CPDs were discovered^16^.

A prior study by our group had taken a tentative look at this topic^16^in protein encoding genes, using the dataset described above^17^. To ensure true disease-causing mutations were considered, a modified version of a score system designed for use in the protein-encoding genes was applied to assess the genotype–phenotype link^18^. Three proven pathogenic point mutations were found across the seven genes of Complex I. Only one of them represented the same amino acid change as in human disease, this being the 3308 T > C^17^.

One explanation for fewer mutations being seen in the protein-encoding genes compared to the mt-tRNA genes is the differential strength of purifying selection on the gene types during the formation of primordial germ cells. Different strengths of selection in this context have been demonstrated in murine models, where variation in the protein-encoding genes is eliminated within a few generations, but variations in the mt-tRNAs persist for far longer^18,20^. Other publications have suggested that pathogenic mutations in protein encoding genes could be masked by supernumerary nuclear proteins, resulting in a stabilization of the protein complexes^20^ in the face of deleterious mutations^21^.

Additional evidence to support the importance of mitochondrial sequence context in the expression and penetrance of pathogenic mtDNA mutations is sought. With a focus on the protein-encoding genes of Complex I, we look for CPDs in more than 900 Metazoan species, representing a more comprehensive phylogenetic range than in past analysis. Wide phylogenetic coverage and a large number of species in the data allowed us to find numerous CPDs for pathogenic mutations in mitochondrial-encoded proteins of Complex I with a “local interactions” approach taken to identify potentially permissive evolutionary events. We suggest that some of these changes might be found among healthy humans as polymorphisms, especially when the evolutionary distance between humans and species with CPDs is short.

## Materials and methods

### Mutation data collection

A dataset of Complex I mutations was generated using variants listed on MitoMap (updated on 04 January 2019), the Mitchell et al. paper, and by running a PubMed search for each of the MTND genes followed by “mutation” and “mitochondria”^2,20^. Variants only reported in association with complex diseases such as Alzheimer’s and Parkinson’s disease were removed from the list of variants from the outset. The list of LHON mutations were taken from the MitoMap database^2^.

We use a modified version of the pathogenicity scoring system used in the context of Complex I mutations to ensure all the variants studied are likely to be associated with disease in humans^17^. The modifications made are in line with those made to the original mt-tRNA diagnostic algorithm^8^. The updated version of the mt-tRNA score system placed a greater emphasis on functional laboratory evidence such as cybrid analysis and single fibre analysis, as these provide the most persuasive evidence of a link between genotype and phenotype^8^. The modified version of the Complex I score system used here  has also changed how conservation is evaluated using an updated tool, moving away from the use of a database employed initially that is no longer updated^22^. Now applying the bioinformatics pathogenicity prediction tool PolyPhen2^23^ to make an assessment of the likelihood that the variant is mildly deleterious.

### Search for CPDs and potential permissive/compensatory substitutions

We took alignments and phylogenetic trees of mitochondrial-encoded Complex I proteins for more than 900 Metazoans from^5,6^ (Supplementary table S1). In order to find species with CPDs, we looked for human pathogenic amino acids in our alignments. If several species carried human pathogenic amino acid at the same site, we estimated the number of substitutions to this amino acid by using our phylogenetic trees with reconstructed ancestral states. Alignment quality at all positions with CPDs was manually inspected.

We looked for potential permissive or compensating substitutions among sites of the same protein chain whose Cβ atoms (Cα for glycine) were closer than 8 Å to Cβ atoms (Cα for glycine) of the site under consideration according to cryo-EM structure of human Complex I (PDB ID: 5XTD)^24^. This distance threshold is frequently used to define amino acid residues that are in contact in folded proteins, including the Critical Assessment of Structure Prediction (CASP) competition^25^. Distances between atoms were calculated with custom BioPython scripts^26^. For each site that was spatially proximal to the CPD site, we checked whether species in our alignment with the CPD contained other non-human amino acids and considered such amino acids as potentially masking variants. We were looking for such variants in all species with CPDs harbouring definitely pathogenic variants, but only in primates for those with probably pathogenic variants or LHON variants.

### Calculation of selective constraints

To check for possible relaxation of negative selection in mitochondrial proteins in clades with CPDs (when substitution to human pathogenic amino acids occurred in the lowest common ancestor of species from this clade), we measured pairwise dN/dS ratio for the protein between two random species from each clade with the CPD using the codeml program of PAML (version 4.6)^27^. When substitutions to the human pathogenic amino acid occurred on the terminal branch, we calculated dN/dS between this species and the closest species without the CPD.

## Results

### Variants classed as definitely pathogenic in humans

**Figure 1:**
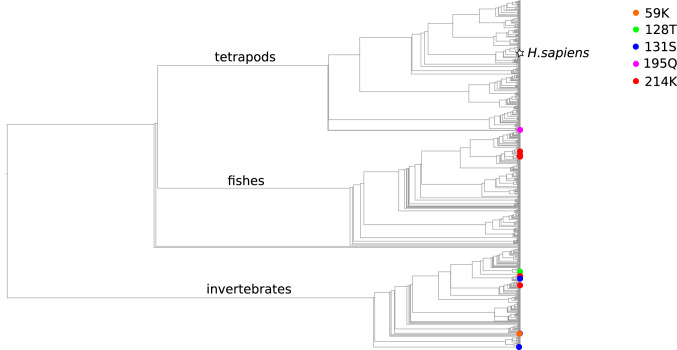
Substitutions to human pathogenic amino acids in sites of ND1 across the cladogram of Metazoa. White star, human branch.

Among the 18 mutations of Complex 1 that are definitely pathogenic after application of the modified scoring system, 11 were found in at least one species from our dataset, and four of them occurred on the phylogenetic tree more than once (Table 1, for species names see Table S2, Supplementary Materials). It should be noted that many of the species with CPDs in the current dataset are distantly related to humans. Twelve substitutions of the amino acids in ND1 that are pathogenic in humans that occurred on our phylogenetic tree were not seen in mammals, with five being seen in vertebrates and seven in invertebrates (see Table 1 and Fig. 1). Overall, among the 28 substitutions to any amino acids that have been reported as pathogenic in humans across all Complex I proteins encoded by mtDNA, only eight took place in vertebrates.

**Table 1:** CPD in mitochondrially encoded Complex I proteins. Variants that are considered in details below are shown in bold.

Gene	Nucleotide mutation	Amino acid mutation	Associated disease	Clades with CPD (class)	Number of substitutions to pathogenic AA on a tree
ND1	m.3481G > A	**E59K**	MELAS, Progressive Encephalopathy	4 mites (Arachnida)	1
	m.3688G > A	A128T	LS	1 butterfly (Insecta)	1
	m.3697G > A	**G131S**	MELAS	5 parasitic flatworms (Cestoda) 2 mites (Arachnida) 1 beetle (Insecta)	3
	m.3890G > A	R195Q	LS, LHON	1 amphibian (Amphibia)	1
	m.3946G > A	**E214K**	MELAS	5 bony fishes (Actinopterygii) 2 insects (Insecta)	6
ND2	m.4681 T > C	L71P	LS	1 bird (Aves)	1
ND3	m.10158 T > C	**S34P**	LS	3 hexapods (Entognatha) 1 sea spider (Pycnogonida) 1 turtle (Reptilia)	4
	m.10197G > A	**A47T**	LS, DYT, LDYT	5 insects (Insecta) 3 spiders (Arachnida) 23 cephalopods (Cephalopoda) 3 turtles (Reptilia)	8
ND5	m.13063G > A	V243I	LS	1 spider (Arachnida)	1
ND6	m.14487 T > C	M63V	LS, DYT	1 insect (Insecta)	1
	m.14600G > A	P25L	LS with sensorineural deafness	1 insect (Insecta)	1

MELAS—Mitochondrial encephalomyopathy, lactic acidosis, and stroke-like episodes; LS—Leigh Syndrome; LHON—Leber's hereditary optic neuropathy; DYT – dystonia; LDYT—Leber's disease and dystonia.

### Some CPDs can occur due to permissive/compensatory substitutions in the same protein

For each site with a CPD we looked for non-human amino acids in sites that are closer than 8 $\AA$ to this site in a spatial structure in order to find amino acid substitutions that could potentially make the human pathogenic amino acids permissible. As the average amino acid identity between Complex 1 proteins of humans and of species with CPDs in our study is 50, we expected that many would carry non-human amino acids at interacting sites. Indeed, only 49 of 103 sites that putatively interact with a CPD-carrying site had the same amino acid in humans. More interestingly, among the remaining 54 sites, 14 had non-human amino acids in all species with CPDs potentially giving us insight into to permissible combinations of amino acids (Table 2).

**Table 2:** Putative permissive substitutions in spatially close sites of the same protein. In the last two columns, there are number of positions, and positions are listed in brackets. Variants that are considered in detail below are shown in bold.

Protein	CPD	Number of putatively interacting sites	Among them with non-human amino acids at least in one species with CPD	Among them with non-human amino acids in all species with CPD
ND1	**59 K**	5	4 (60, 61, 216, 217)	3 (60, 216, 217)
	128 T	17	4 (119, 130, 132, 213)	-
	**131S**	14	10 (127, 128, 129, 130, 132, 133, 135, 200, 201, 203)	6 (128, 129, 130, 132, 135, 200)
	195Q	10	4 (194, 196, 198, 273)	-
	**214 K**	10	4 (61, 66, 213, 216)	2 (61, 213)
ND2	71P	9	7 (68,69,70,72,73,74,75)	-
ND3	**34P**	3	1 (35)	0
	**47 T**	3	2 (45,46)	1(46)
ND5	243I	12	4(155,157,239,247)	-
ND6	25L	9	6 (24,26,27,28,68,74)	-
	63 V	8	5 (58,59,64,65,66)	-

We looked in more detail at potential CPDs (or masking variants) that were found in more than one species in our dataset.

At site 59 of ND1, Glutamic Acid (E) is the major human variant, this variant is highly conserved occupying this site in 99% of species in our dataset. Lysine (K), which is pathogenic in humans, was found only in one clade consisting of four species of mites. There are five sites that potentially interact with site 59 of ND1. Among them, three sites (60, 216, 217) contain a non-human amino acid, and this is in all four mite species with the CPD. In two of these sites (60, 216) the human amino acid is the major amino acid across the phylogenetic tree being found in 70% and 69% of species, respectively. In contrast, the amino acids at these sites in mites with CPDs are minor, seen in a very small number of species: 0.4% and 0.4% for sites 60 and 216 respectively. And at site 217, both the human and potentially permissive amino acids are minor alleles, but comparatively frequent, being found in 29% and 20% of species, respectively. The E59K human variant changes the charge of the residue, but there are no charge-changing substitutions in spatial proximity of site 59 of ND1 in species with the CPD.

Considering site 131 of ND1, the human pathogenetic substitution to Serine (S) was seen to occur three times in our dataset, in five parasitic flatworms, two mites and one beetle. This position is highly conserved throughout evolution, and its homologous site in the NuoH protein of *E.coli*, carrying the wild-type amino acid, was shown to play an essential role in stability of Complex I^28^. Among 14 sites that are in close spatial proximity to site 131 of ND1, 10 contain non-human amino acids in at least one of three clades with the CPD. At position 135 of ND1, beetles and worms have Cysteine (C) due to independent substitution events, and the mites have Valine (V). Both amino acids are very rare across the phylogeny, being seen in 0.3% and 0.25% of species respectively. At positions 201 and 203, which are located far from site 131 in primary structure, but close in 3D structure, mites and worms with 131S have different but rare (no more than in 3% of species from the dataset) non-human amino acids. Interestingly, worms and mites are among species with the highest fraction of rare variants (that are found in less than 10 species from our dataset) amino acids in their ND1 gene. As parasitic flatworms live in hypoxic conditions inside their hosts, the selection that acts on their OXPHOS genes might be relaxed. The ratio of the rates of non-synonymous to synonymous substitutions (dN/dS) of ND1 was 0.18 between Pork tapeworm (*Taenia solium)* and Asian tapeworm (*Taenia asiatica)*, which was twice as high as that seen between humans and gorillas. Nonetheless these rates were still far less than one, suggesting that the protein is still under negative selection.

The ND1:214 K variant was seen to occur six times in our dataset, with five species of fish, one species of fly and one beetle carrying Lysine (K), thus making K the second most common amino acid in the site after the ‘wild-type’ Glutamic Acid I. Among 10 positions structurally close to site 214, four contained non-human amino acids, and two sites, 61 and 213, had non-human amino acids in all species with the 214 K pathogenic human variant: Valine (V), Isoleucine (I) and Threonine (T) in site 61 and Valine (V) in site 213. The 213 V variant is an ancestral amino acid for the tree and is the most prevalent amino acid in this site, being seen in 88% of species. In contrast, the human amino acid Isoleucine (I) is seen in 9% of species. There were 34 substitutions leading to this variant including a substitution in the lowest common ancestor of monkeys. Thus, it is possible that I213 creates a pathogenic potential for the K at site 214. Interestingly, homologous positions of the NuoH protein of *E.coli* carry the same amino acids as human ND1 does: I227 (homologous to I213 in human) and E228 (homologous to E214 in human), and an E228K mutation leads to assembly of practically non-functional enzyme in E.coli^29^.

At site 34 of ND3, the human amino acid Serine (S) is not a major amino acid seen on the tree being found in only 8.5% of species, and this site is not highly conserved: there are 14 amino acids that occupy it in more than one species. This could mean that many amino acids are benign in ND3:34 simultaneously. Alternatively, it could mean that the fitness landscape of this site changes frequently, and the occurrence of human pathogenic amino acid 34P as a wild-type amino acid in other species is evidence to support this notion. There are only three ND3 sites structurally close to site 34, none of them having non-human amino acids in the five species with the CPD.

We found the human wild-type variant ND3:47A in 93%, and the pathogenic human variant ND3:47 T in 1% of 2766 species where this position was covered in our dataset. Only one of three contacting sites carried the non-human amino acids in all 34 species with this CPD. It was not only one amino acid, but 10 different amino acids. Other contacting sites carried non-human amino acids in 26 (site 45) and 0 (site 48) of 34 species with CPDs. Thus, substitutions in one or several contacting sites may mask pathogenic effect of ND3:47A. Both sites 34 and 47 of ND3 are included in a loop between trans-membrane regions 1 and 2 (so-called TMH1-2 loop), which was shown to be critical for proton pumping^28,30^.

### Probably pathogenic amino acids

Besides mutations with “Pathogenic” status, “Probably pathogenic” amino acids also have strong evidence for association with disease in humans, especially if they have functional evidence to support their categorization. We found 10 probably pathogenic mutations as a ‘wild-type’ allele in at least one species used in our study (Table 3, for species names see Table S3, Supplementary Materials). Of these, we focused on site ND5:398 (13528A > G)^36^, where the human pathogenic variant was prevalent in non-human species, and even represented the wild-type allele in some primates. Interestingly, most of the primates with the pathogenic change at site ND5:398 belonged to *Cercopithecinae* subfamily, or old world monkeys. This group shares ~ 80% amino acid identity with humans in the ND5 gene. The variants have functional evidence of pathogenicity in humans^31^.

**Table 3:** Probably pathogenic human variants in non-human species. Variants that are considered in detail below are shown in bold.

Gene	Mutation	Protein mutation	Associated disease	Number of species	Number of substitutions	Closest species to human (class)
**ND1**	3376G > A	E24K	LHON, MELAS Overlap	5	5	Bony fishes (Actinopterygii)
	3380G > A	R25Q	MELAS	3	3	Insects (Insecta)
	3388C > A	L28M	Non-syndromic Hearing Loss	7	3	Reptiles (Reptilia)
	3928G > C	V208L	LS	1	1	Lancelets (Leptocardii)
**ND3**	10254G > A	D66N	LS	1	1	Crustaceans (Malacostraca)
	11240C > T	L161F	LS	8	4	Insects (Insecta)
**ND5**	**13528A > G**	**T398A**	**LHON-like, MELAS**	**545**	**8**	**Primates (Mammalia)**
	**13565C > T**	**S410F**	**MELAS**	**68**	**13**	**Primates (Mammalia)**
**ND6**	14439G > A	P79S	LS	2	1	Bony fishes (Actinopterygii)
	14453G > A	A74V	MELAS	6	3	Carnivorans (Mammalia)

### ND5: T398A

**Figure 2:**
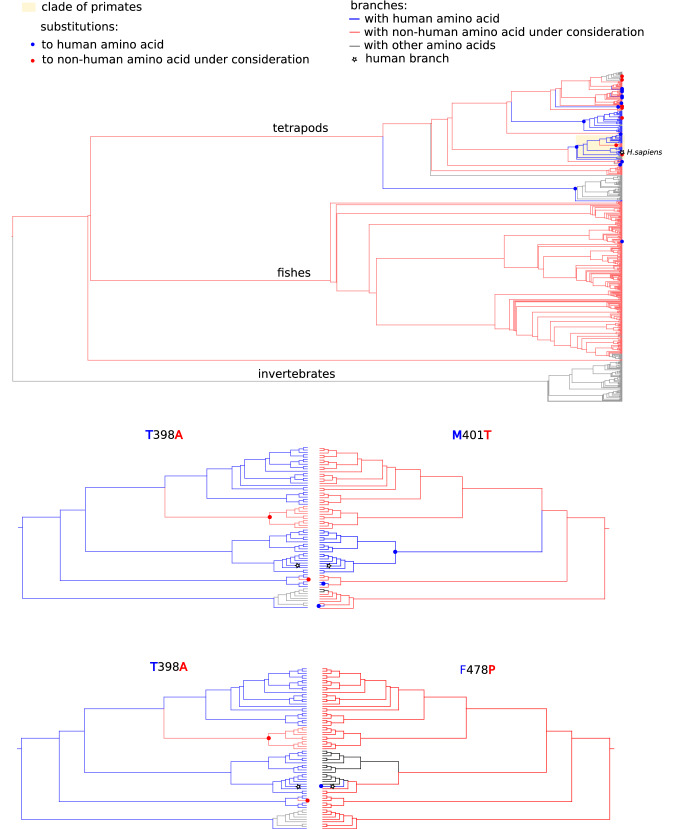
Substitutions to human wild type (T, blue) and pathogenic (A, red) amino acids on site 398 of ND5 on the cladogram. The cladogram is colored according to occupying amino acid: coral, A; blue, T; black, other amino acids. Primate clade has yellow background. Upper cladogram show the whole phylogenetic range considered, and other cladograms show primate clade. White star, Homo sapiens branch.

The human pathogenic amino acid Alanine (A) is the most prevalent amino acid in this site, found in 59% of species in our dataset, and human amino acid Threonine (T) is found in 14% of species. The A398T substitution occurred in the lowest common ancestor (LCA) of primates, and two T398A reversions occurred: one in the Squirrel monkey, and one in the root of *Cercopithecinae* subfamily that is represented by 10 species in our dataset (Fig. 2). Interestingly, 129 of 130 species with 398 T belong to the *Terapoda* clade, a clade consisting of four-limbed animals.

Among the 14 ND5 sites that are closer than 8 Å to site 398, five sites carried non-human amino acids in at least one primate species with the CPD. Of these, three sites (394, 401 and 478) carried non-human amino acids in all 11 primate species with the CPD (Fig. 2). In sites 401 and 478, these non-human amino acids are seen as the most prevalent amino acids in corresponding sites across the dataset (76% and 72%, for sites 401 and 478, respectively), and in site 394, this amino acid is the second most prevalent (41% of species). Among 545 species with the human pathogenic amino acid (A) in site 398, only five contained human amino acid Methionine (M) in site 401. Furthermore, none contained human amino acids Histidine (H) in site 394 and Phenylalanine (F) in site 478. This suggests that the combination of human variants H394, M401 and F478 with human pathogenic variant A398 may be undesirable.

### Human LHON variants can be met in closely related species

Leber’s Hereditary Optic Neuropathy (LHON) is a debilitating disease which causes loss of retinal ganglion cells within the central retina and subsequent degeneration of the optic nerve. Patients develop acute blindness within six weeks of symptom onset. LHON-causing amino acid variants do not score highly in the current pathogenicity scoring systems, including our updated version. There are a number of reasons for this, including the possibility for LHON causing mutations to be present as homoplasmic variants in unaffected individuals. Thus we looked for CPDs for the “top-19” nucleotide variants associated with LHON according to MitoMap ^2^ that lead to 18 different amino acids. The three most common (m.11778G > A, m.3460G > A and m.14484 T > C) of the 18 variants are thought to cause > 85% of LHON cases. Dramatically, we found no species with these amino acids in our data (Table D). Among the remaining 15 mitochondrial variants considered to have good evidence for association with LHON on the MitoMap database, five were associated with other syndromes in addition to LHON, and these variants were previously analyzed in this paper as “pathogenic” or “probably pathogenic” (Table 4, for species names see Table S3, Supplementary Materials). We have found eight of the 10 remaining amino acid changes in at least one metazoan species form our datasets. For one site, (ND4L:65) the human LHON amino acid (A) was the major variant in a tree, and for two sites (ND1:132, ND6:58), LHON amino acids were found in primate species. We took a closer look at these variants, specifically looking at local interactions to find potential permissive substitutions in these close human relatives.

**Table 4:** Human LHON variants in non-human species. Variants that are considered in detail below are shown in bold.

Gene	Mutation	Protein mutation	Number of species	Number of substitutions	Closest clade to human
**ND1**	**m.3700G > A**	**A132T**	**4**	**4**	**Primates (Mammalia)**
	m.3733G > A	E143K	2	1	Bony fishes (Actinopterygii)
	m.4171C > A	L289M	24	6	Amphibians (Amphibia)
**ND4L**	**m.10663 T > C**	**V65A**	**630**	**16**	**Reptiles (Reptilia)**
**ND6**	m.14482C > A(G)	M64I	1	1	Birds (Aves)
	m.14495A > G	L60S	1	1	Bony fishes (Actinopterygii)
	**m.14502 T > C**	**I58V**	**237**	**34**	**Primates (Mammalia)**
	m.14568C > T	G36S	124	15	Birds (Aves)

### ND1:A132T

Human LHON variant T132 was found in four species: three vertebrates and one invertebrate, among them was Northwest Bornean orangutan (*Pongo pygmaeus pygmaeus*), while its close relative Sumatran orangutan (*Pongo abelii*) carried the human variant A132. Variant T132 has already been considered in Bornean orangutan^32^. The estimated divergence time of Bornean and Sumatran orangutans is between 400 000—1Mya, and site 132 is among 15 of 318 ND1 amino acids that differ between Bornean and Sumatran orangutans^33^.

**Figure 3:**
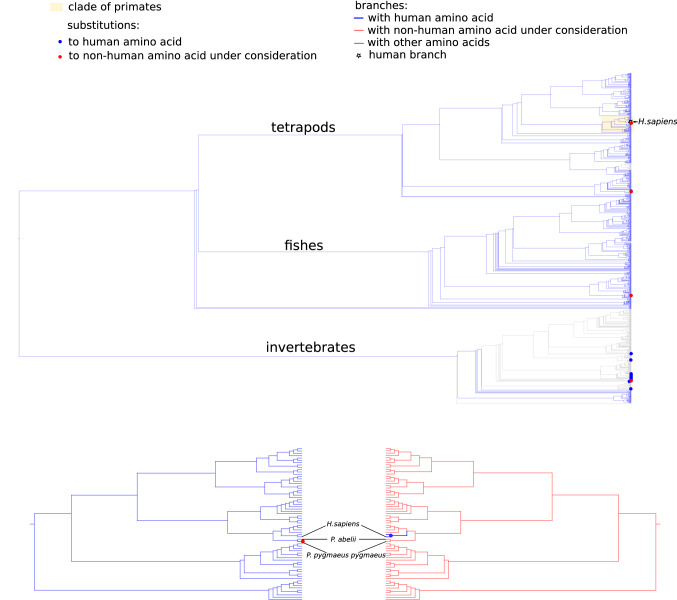
Substitutions to A (blue dot) and T (red dot) in sites 132 and 201 of ND1 across the dataset (top) and on a clade of Primates (bottom). Blue and red branches are occupied by A and T, respectively. White star—Homo sapiens branch.

Sixteen amino acid positions of ND1 were closer than 8 Å to ND1:132, six of which were further than five positions from it in a primary structure, and only site 201 carried non-human amino acid in Bornean orangutan. Both Bornean and Sumatran orangutans carried the same non-human variant T201 (Table 5, Fig. 3). Of 67 primates in our dataset, A201 was found only in humans and gorillas (common ancestry). T201 is a major amino acid in vertebrates. A201T substitutions occurred in the LCA of vertebrates, and it was 15 T201A reversions that led to 20 vertebrate species with A201. Thus, amino acid A201 could make T132 deleterious in humans. Sites 132 and 201 both belong to non-membrane regions of the protein, facing the same side of the inner mitochondrial membrane. These loops are highly conserved across the tree of life and play an important role in Complex I assembly, and position 132 carries the same amino acid A in both humans and *E.coli*.^28^.

**Table 5:** Co-occurrence of Alanine (A) and Threonine (T) in sites 132 and 201 of ND1 in our dataset.

Variant	132	201	Number of species	Species with CPD
Human, gorilla	A	A	185	-
Bornean orangutan	T	T	3	Pongo pygmaeus pygmaeus, primates Alepocephalus tenebrosus,fishes Onychodactylus fischeri,salamanders
Sumatran orangutan	A	T	2666	-
LHON	T	A	1	Onychiurus orientalis, springtails

### ND4L:V65A

Human wild-type allele Valine (V) and human pathogenic amino acid Alanine (A) are the two most prevalent amino acids in the dataset (31% and 36% of species, respectively). The closest animals to humans with Alanine (A) in position 65 are reptiles. Human amino acid Valine (V) is the major amino acid in mammals being found in 462 of 464 mammalian species in our dataset. The human LHON associated variant A65 is a major amino acid in fish and is also prevalent in birds and reptiles. Substitution A65V that led to V in humans occurred in the LCA of mammals. Thus, the LHON-causing V65A mutation in humans is an undesired reversion to the mammalian ancestral state.

Although just one C-T transition suffices to mutate A and V to one another, very few substitutions between these amino acids occurred in a tree (1 of 13 substitutions from V are V > A, and only 2 of 48 substitutions from A are A > V). This suggests that one of these amino acids might be unfavorable in lineages where the other one is prevalent. This is consistent with differences in their physicochemical properties: According to ranking of amino acids by physicochemical similarity based on the Miyata matrix^34^, V has rank 6 for A, and A has rank 9 for V.

### ND6:I58V

**Figure 4:**
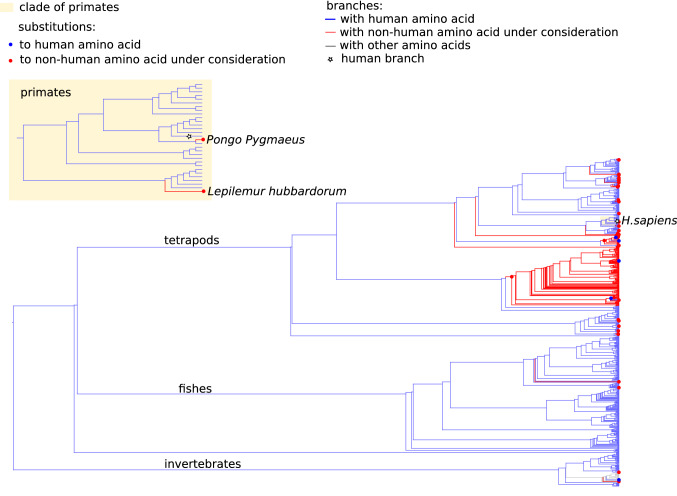
Substitutions to I (human normal variant, blue dot) and V (LHON variant, red dot) in site 58 of ND6 across the dataset and on a clade of Primates (yellow rectangle). Blue and red branches are occupied by I and V, respectively. White star—Homo sapiens branch.

There was a total of 237 species, mostly amniotes, that carried human LHON variant I58V in ND6. Among them two were primates: *Pongo pygmaeus* (Bornean orangutan) and *Lepilemur hubbardorum* (Hubbard's sportive lemur) (Fig. 4). The Amino acid Isoleucine (I) is ancestral and the most prevalent variant in site 58 on the tree. Among 35 58 V substitutions in our phylogenetic tree, 34 were I58V substitutions, 22 of which occurred in mammals, including substitutions in Bornean orangutan and Hubbard’s sportive lemur.

Among the seven sites of ND6 that are closer than 8 Å to position 58, only one had a non-human amino acid in Bornean orangutan (V54 instead of M54), and no sites carried non-human amino acids in Hubbard’s sportive lemur. In site 54 of ND6, orangutan variant V was a major amino acid in the whole phylogenetic tree, but human variant M was a major variant in mammals. Only two M54V substitutions occurred in mammals, one of which was in Bornean orangutan.

Site ND6:58 is a part of the B/C hydrophobic domain of the protein, which is conserved in humans^35^. Given the similarity of 59% between ND6 of humans and Hubbard's sportive lemur, it is rather surprising to find no non-human amino acids in sites involved in local interactions with site 58, assuming the uniform distribution of sites with different evolutionary rates along the protein (hypergeometrical probability of observing no non-human amino acids in seven sites = 0.02). Therefore, the structural region with the CPD is evolutionarily conserved between the two species more than the protein on average.

## Discussion

There has been much speculation about the importance of mtDNA haplogroup context in the expression and penetrance of mtDNA mutations^12^. Here we take the most detailed look yet at possible importance of sequence context in the pathogenicity of Complex I mutations considering intra peptide interactions of mtDNA encoded subunits of complex 1.

Firstly, we re-examined the algorithm to assign pathogenicity to Complex I mutations in line with the re-assessment conducted for the mt-tRNA mutations^8^. Principally, we increased the weighting placed on functional laboratory evidence, as this provides the best link between genotype and phenotype. All these efforts ensured that the mutations we considered were disease causing or had a high probability of being associated with mitochondrial disease.

CPDs that are found in primates represent the most intriguing cases, as these variants are more likely to persist as polymorphisms in human populations, potentially being responsible for the variability in consequences of the same mutations in different people. In support of this, ND6:I58V LHON variant is a normal variant of Bornean orangutan, but not for its close relative, Sumatran orangutan. Simultaneously, only Bornean orangutan has a non-human amino acid in potentially interacting site 54, which likely masks the deleterious effect of V58. These two species are able to produce reproductively viable progeny, and their separation is still under debate, nevertheless, the V58 variant is likely to be pathogenic in Sumatran orangutan while benign in Bornean orangutan. Interestingly, the 14502 T > C nucleotide substitution that results in I58V can been seen in 186 publicly available human sequences without a disease report^2^. This nucleotide substitution is a haplogroup marker for the R8b2, P7, X2a, N11a and M10 lineages. With the exception of X2a, these are all non-European lineages^3^.

The occurrence of LHON amino acids in a primate species (*Lepilemur hubbardorum*) with human variants in contacting residues is another interesting finding. There was only one Lepilemur species in our tree, but among 16 species of this genus with an ND6 sequence available in UniProt, 11 carried human LHON variant V, and 3 carried human wild-type variant I. Central vision loss might be much more deleterious for primates in the wild than for humans, thus there might be stronger selection against such variants even if the penetrance is less than 100%. As Lepilemurs are nocturnal animals, some aspects of vision have different importance for them than for humans^36^. For example, loss of color vision—one of LHON symptoms—may not be deleterious for them. Nevertheless, complete loss of central vision—the most common LHON outcome, would be fatal for these animals which move by long jumps between tree branches. Specific features of LHON, such as gender bias, existence of unknown triggers that lead to disease progression in early adulthood, and rare cases of vision recovery, make LHON an enigmatic disease^37^. Thus, Lepilemurs that carry human LHON variants without apparent compensations, have a potential to shed light on triggers of LHON progression, as such triggers seem to be absent in this genus.

In some species, CPDs could become possible because of more hypoxic environments, as hypoxia is suggested to decrease penetrance of some mitochondrial mutations^38,39^. We found that this is possibly the case with ND1:S131 in parasitic flatworms. Finally, given the highly variable nature of the species that we have studied, it should be considered that diet might impact upon the pathogenicity, or not, of some of the mutations, as has been demonstrated in model species by other groups using *Drosophila melanogaster*^39^.

It must also be remembered that, in some cases, variation in other genes can lead to the reversal of disease phenotypes resulting from mtDNA mutations, with such a change in the expression of the homoplasmic m.14674 T > C mutation being a well-studied example^21^. Thus, both intra- and inter-masking variants for CPDs are possible. In this paper, we have only investigated the role of intra-protein variation in the existence of CPDs.

In summary, our work has shown that sequence context is important in the expression of mutations in Complex I genes, with variants being permissible in specific contexts, and disallowed in others. Thus, the notion might not always hold that if a variant is seen as a population or haplogroup marker, it should be discounted as having a role in disease. The distinction between mild pathogenic mtDNA mutations and population polymorphisms can be difficult to define and might change in environmental as well as sequence context^ 16,38,40^.

## Supplementary Information

Supplementary Tables.
