# Supplementary material for: A broad comparative genomics approach to understanding the pathogenicity of Complex I mutations
Source: Sci Rep. 2021 Oct 1;11:19578. doi: 10.1038/s41598-021-98360-7 (PMC8486755; doi:10.1038/s41598-021-98360-7)
Supplement: Supplementary file 1 — Supplementary Tables. [file 41598_2021_98360_MOESM1_ESM.docx]

**Supplementary materials**

Supplementary table S1. Dataset

| **Gene** | **Number of species** | **Amino acids per site** | **Substitutions per site** |
| --- | --- | --- | --- |
| ND1 | 2013 | 8.7 | 92.9 |
| ND2 | 5765 | 10.2 | 259.6 |
| ND3 | 2766 | 9.7 | 182.3 |
| ND4 | 2007 | 9.1 | 127.1 |
| ND4L | 1759 | 11.3 | 139.3 |
| ND5 | 926 | 7.9 | 57.6 |
| ND6 | 996 | 10.5 | 76.6 |

Supplementary table S2. Pathogenic mutations and species where they were found in multi-species protein alignments.

| ***gene*** | ***mutation*** | ***amino acid change*** | ***species* with *CPD* *(NCBI IDs)*** | ***number of species with CPD*** | ***number of substitutions to pathogenic AA on a tree*** |
| --- | --- | --- | --- | --- | --- |
| **ND1** | 3481G>A | **E59K** | 50023 - [Panonychus citri](https://www.ncbi.nlm.nih.gov/Taxonomy/Browser/wwwtax.cgi?id=50023) (mite)  50024 - [Panonychus ulmi](https://www.ncbi.nlm.nih.gov/Taxonomy/Browser/wwwtax.cgi?id=50024) (mite)  93129 - [Tetranychus cinnabarinus](https://www.ncbi.nlm.nih.gov/Taxonomy/Browser/wwwtax.cgi?id=93129) (mite)  32264 - [Tetranychus urticae](https://www.ncbi.nlm.nih.gov/Taxonomy/Browser/wwwtax.cgi?id=32264) (mite) | 4 | 1 |
|  | 3688G>A | **A128T** | 501369 - [Troides aeacus](https://www.ncbi.nlm.nih.gov/Taxonomy/Browser/wwwtax.cgi?id=501369) (butterfly) | 1 | 1 |
|  | 3697G>A | **G131S** | 6206 - [Taenia saginata](https://www.ncbi.nlm.nih.gov/Taxonomy/Browser/wwwtax.cgi?id=6206) (flatworm)  6204 - [Taenia solium](https://www.ncbi.nlm.nih.gov/Taxonomy/Browser/wwwtax.cgi?id=6204) (flatworm)  60517 - [Taenia asiatica](https://www.ncbi.nlm.nih.gov/Taxonomy/Browser/wwwtax.cgi?id=60517) (flatworm)  6210 - [Echinococcus granulosus](https://www.ncbi.nlm.nih.gov/Taxonomy/Browser/wwwtax.cgi?id=6210) (flatworm)  519352 - [Echinococcus canadensis](https://www.ncbi.nlm.nih.gov/Taxonomy/Browser/wwwtax.cgi?id=519352) (flatworm)  32264 - [Tetranychus urticae](https://www.ncbi.nlm.nih.gov/Taxonomy/Browser/wwwtax.cgi?id=32264) (mite)  93129 - [Tetranychus cinnabarinus](https://www.ncbi.nlm.nih.gov/Taxonomy/Browser/wwwtax.cgi?id=93129) (mite)  71223 - [Pyrocoelia rufa](https://www.ncbi.nlm.nih.gov/Taxonomy/Browser/wwwtax.cgi?id=71223) (beetle) | 8 | 3 |
|  | 3890G>A | **R195Q** | 161274 - [Bombina maxima](https://www.ncbi.nlm.nih.gov/Taxonomy/Browser/wwwtax.cgi?id=161274) (toad) | 1 | 1 |
|  | 3946G>A | **E214K** | 166768 - [Typhlogobius californiensis](https://www.ncbi.nlm.nih.gov/Taxonomy/Browser/wwwtax.cgi?id=166768) (fish)  166755 - [Periophthalmus barbarus](https://www.ncbi.nlm.nih.gov/Taxonomy/Browser/wwwtax.cgi?id=166755) (fish)  308075 - [Leptophilypnus fluviatilis](https://www.ncbi.nlm.nih.gov/Taxonomy/Browser/wwwtax.cgi?id=308075) (fish)  166760 - [Pseudapocryptes elongatus](https://www.ncbi.nlm.nih.gov/Taxonomy/Browser/wwwtax.cgi?id=166760) (fish)  166754 - [Odontamblyopus rubicundus](https://www.ncbi.nlm.nih.gov/Taxonomy/Browser/wwwtax.cgi?id=166754) (fish)  7238 - [Drosophila sechellia](https://www.ncbi.nlm.nih.gov/Taxonomy/Browser/wwwtax.cgi?id=7238) (fly)  184539 - [Crioceris duodecimpunctata](https://www.ncbi.nlm.nih.gov/Taxonomy/Browser/wwwtax.cgi?id=184539) (beetle) | 7 | 6 |
|  | 3949T>C | **Y215H** | - | - | - |
| **ND2** | 4681T>C | **L71P** | 272050 - [Tringa erythropus](https://www.ncbi.nlm.nih.gov/Taxonomy/Browser/wwwtax.cgi?id=272050) (bird) | 1 | 1 |
| **ND3** | 10158T>C | **S34P** | 187695 - [Sinella curviseta](https://www.ncbi.nlm.nih.gov/Taxonomy/Browser/wwwtax.cgi?id=187695) (springtail)  187623 - [Cryptopygus antarcticus](https://www.ncbi.nlm.nih.gov/Taxonomy/Browser/wwwtax.cgi?id=187623) (springtail)  296598 - [Japyx solifugus](https://www.ncbi.nlm.nih.gov/Taxonomy/Browser/wwwtax.cgi?id=296598) (Dipluran)  136195 - [Nymphon gracile](https://www.ncbi.nlm.nih.gov/Taxonomy/Browser/wwwtax.cgi?id=136195) (seq spider)  286009 - [Malacochersus tornieri](https://www.ncbi.nlm.nih.gov/Taxonomy/Browser/wwwtax.cgi?id=286009) (turtle) | 5 | 4 |
|  | 10191T>C | **S45P** | - | - | - |
|  | 10197G>A | **A47T** | 61727 - [Onychoteuthis compacta](https://www.ncbi.nlm.nih.gov/Taxonomy/Browser/wwwtax.cgi?id=61727) (cephalopod)  309596 - [Gonatus pyros](https://www.ncbi.nlm.nih.gov/Taxonomy/Browser/wwwtax.cgi?id=309596) (cephalopod)  392295 - [Onychoteuthis borealijaponica](https://www.ncbi.nlm.nih.gov/Taxonomy/Browser/wwwtax.cgi?id=392295) (cephalopod)  61679 - [Ommastrephes bartramii](https://www.ncbi.nlm.nih.gov/Taxonomy/Browser/wwwtax.cgi?id=61679) (cephalopod)  55288 - [Vampyroteuthis infernalis](https://www.ncbi.nlm.nih.gov/Taxonomy/Browser/wwwtax.cgi?id=55288) (cephalopod)  31210 - [Sepia esculenta](https://www.ncbi.nlm.nih.gov/Taxonomy/Browser/wwwtax.cgi?id=31210) (cephalopod)  61751 - [Thysanoteuthis rhombus](https://www.ncbi.nlm.nih.gov/Taxonomy/Browser/wwwtax.cgi?id=61751) (cephalopod)  6637 – Todarodes pacificus (cephalopod)  346249 - [Dosidicus gigas](https://www.ncbi.nlm.nih.gov/Taxonomy/Browser/wwwtax.cgi?id=346249) (cephalopod)  6610 - [Sepia officinalis](https://www.ncbi.nlm.nih.gov/Taxonomy/Browser/wwwtax.cgi?id=6610) (cephalopod)  34570 - [Sepioteuthis lessoniana](https://www.ncbi.nlm.nih.gov/Taxonomy/Browser/wwwtax.cgi?id=34570) (cephalopod)  94907 - [Gonatus californiensis](https://www.ncbi.nlm.nih.gov/Taxonomy/Browser/wwwtax.cgi?id=94907) (cephalopod)  61685= 2053935 - [Ancistrocheirus lesueurii](https://www.ncbi.nlm.nih.gov/Taxonomy/Browser/wwwtax.cgi?id=2053935) (cephalopod)  34553 - [Sthenoteuthis oualaniensis](https://www.ncbi.nlm.nih.gov/Taxonomy/Browser/wwwtax.cgi?id=34553) (cephalopod)  309546 - [Eucleoteuthis luminosa](https://www.ncbi.nlm.nih.gov/Taxonomy/Browser/wwwtax.cgi?id=309546) (cephalopod)  294704 - [Berryteuthis magister](https://www.ncbi.nlm.nih.gov/Taxonomy/Browser/wwwtax.cgi?id=294704) (cephalopod)  61699 - [Chtenopteryx sicula](https://www.ncbi.nlm.nih.gov/Taxonomy/Browser/wwwtax.cgi?id=61699) (cephalopod)  256136 - [Architeuthis dux](https://www.ncbi.nlm.nih.gov/Taxonomy/Browser/wwwtax.cgi?id=256136) (cephalopod)  61704 - [Cranchia scabra](https://www.ncbi.nlm.nih.gov/Taxonomy/Browser/wwwtax.cgi?id=61704) (cephalopod)  1051066 - [Doryteuthis opalescens](https://www.ncbi.nlm.nih.gov/Taxonomy/Browser/wwwtax.cgi?id=1051066) (cephalopod)  559548 - [Pterygioteuthis giardi](https://www.ncbi.nlm.nih.gov/Taxonomy/Browser/wwwtax.cgi?id=559548) (cephalopod)  6625 - [Watasenia scintillans](https://www.ncbi.nlm.nih.gov/Taxonomy/Browser/wwwtax.cgi?id=6625) (cephalopod)  72292 - [Onykia robusta](https://www.ncbi.nlm.nih.gov/Taxonomy/Browser/wwwtax.cgi?id=72292) (cephalopod)  74934 - [Sacalia quadriocellata](https://www.ncbi.nlm.nih.gov/Taxonomy/Browser/wwwtax.cgi?id=74934) (turtle)  74926 [Mauremys mutica](https://www.ncbi.nlm.nih.gov/Taxonomy/Browser/wwwtax.cgi?id=74926) (turtle)  204965 - [Cuora flavomarginata](https://www.ncbi.nlm.nih.gov/Taxonomy/Browser/wwwtax.cgi?id=204965) (turtle)  118624 - [Phalangium opilio](https://www.ncbi.nlm.nih.gov/Taxonomy/Browser/wwwtax.cgi?id=118624) (spider)  109461 - [Varroa destructor](https://www.ncbi.nlm.nih.gov/Taxonomy/Browser/wwwtax.cgi?id=109461) (mite)  58767 - [Mastigoproctus giganteus](https://www.ncbi.nlm.nih.gov/Taxonomy/Browser/wwwtax.cgi?id=58767) (whip scorpion)  633874 - [Mordella atrata](https://www.ncbi.nlm.nih.gov/Taxonomy/Browser/wwwtax.cgi?id=633874) (beetle)  130591 - [Lycorma delicatula](https://www.ncbi.nlm.nih.gov/Taxonomy/Browser/wwwtax.cgi?id=130591) (bug)  420844 - [Gampsocleis gratiosa](https://www.ncbi.nlm.nih.gov/Taxonomy/Browser/wwwtax.cgi?id=420844) (cricket)  488323 - [Elimaea cheni](https://www.ncbi.nlm.nih.gov/Taxonomy/Browser/wwwtax.cgi?id=488323) (cricket)  299218 - [Nesomachilis australica](https://www.ncbi.nlm.nih.gov/Taxonomy/Browser/wwwtax.cgi?id=299218) (insect) | 34 | 8 |
| **ND4** | 11777C>A | **R340S** | - | - | - |
| **ND5** | 13063G>A | **V243I** | 130930 - [Habronattus oregonensis](https://www.ncbi.nlm.nih.gov/Taxonomy/Browser/wwwtax.cgi?id=130930) (spider) | 1 | 1 |
|  | 13094T>C | **V253A** | - | - | - |
|  | 13513G>A | **D393N** | - | - | - |
|  | 13514A>G | **D393G** | - | - | - |
| **ND6** | 14459G>A | **A72V** | - | - | - |
|  | 14487T>C | **M63V** | 546486 - [Contacyphon sp. BT0012](https://www.ncbi.nlm.nih.gov/Taxonomy/Browser/wwwtax.cgi?id=546486) (insect) | 1 | 1 |
|  | 14600G>A | **P25L** | 7160 - [Aedes albopictus](https://www.ncbi.nlm.nih.gov/Taxonomy/Browser/wwwtax.cgi?id=7160) (insect) | 1 | 1 |

Supplementary table S3. Probably pathogenic mutations and species where they were found in multi-species protein alignments.

| *gene* | *mutation* | *amino acid change* | *species* with *CPD* *(NCBI IDs)* | *number of species with CPD* | *number of substitutions to pathogenic AA on a tree* |
| --- | --- | --- | --- | --- | --- |
| ND1 | 3310C>T | **P2S** | BAD ALIGNMENT |  |  |
|  | 3376G>A | **E24K** | 155694 - Eurydice pulchra (isopod)  150319 - Ptereleotris zebra (fish)  459531 - Atelura formicaria (nicoletiid)  575843 - Valentia hoffmanni (bug)  7160 - Aedes albopictus (mosquito) | 5 | 5 |
|  | 3380G>A | **R25Q** | 53568 - Tripteroides bambusa (mosquito)  92525 - Asellus aquaticus (isopod)  583350 - Onisimus nanseni (amphipod) | 3 | 3 |
|  | 3388C>A | **L28M** | 509074 - Hypsiglena ochrorhyncha klauberi (snake)  509065 - Hypsiglena chlorophaea chlorophaea (snake)  8496 - Alligator mississippiensis (reptile)  46305 - Hypsiglena torquata (snake)  533186 - Hypsiglena sp. DGM-2008 (snake)  509078 - Hypsiglena slevini (snakes)  291262 - Bolitoglossa n. sp. RLM-2004 (salamander) | 7 | 3 |
|  | 3928G>C** | **V208L** | 7740 - Branchiostoma lanceolatum, amphioxus (lancelet) | 1 | 1 |
| ND3 | 10254G>A | **D66N** | 79674 - Macrobrachium rosenbergii (crustacean) | 1 | 1 |
| ND4 | 11232T>C | **L158P** | - |  | 0 |
|  | 11240C>T | **L161F** | 37777 - Saturnia boisduvalii (moth)  7121 - Antheraea yamamai (moth)  7119 - Antheraea pernyi (moth)  364026 - Eriogyna pyretorum (moth)  267182 - Thrinaconyx fumosus (mantid)  627779 - Thrinaconyx kirschianus (mantid)  444932 - Amantis biroi (mantid)  34615 - Ixodes persulcatus (mite) | 8 | 4 |
| ND5 | 12338T>C | **M1T** | - | 0 | 0 |
|  | 12706T>C | **F124L** | - | 0 | 0 |
|  | 13042G>A | **A236T** | - | 0 | 0 |
|  | 13046T>C | **M237T** | - | 0 | 0 |
|  | 13084A>T | **S250C** | - | 0 | 0 |
|  | 13511A>T | **K392M** | - | 0 | 0 |
|  | 13528A>G | **T398A** | 545 of 926 in a tree  among them 11 primates:  9521 – Saimiri sciureus (common squirrel monkey)  60711 – Chlorocebus sabaeus (green monkey)  60710 - Chlorocebus pygerythrus (vervet)  60712 - Chlorocebus tantalus (tantalus monkey)  9534 - Chlorocebus aethiops (drivet)  9557 - Papio hamadryas (hamadryas baboon)  9565 - Theropithecus gelada (gelada)  9546 Macaca sylvanus (Barbary ape)  54602 - Macaca thibetana (Pere David's macaque)  9541 - Macaca fascicularis (crab-eating macaque)  9544 - Macaca mulatta (Rhesus monkey) | 545 | 8 (A is an ancestral and most prevalent AA in this site)  2 of them in primates |
|  | 13565C>T | **S410F** | 68 of 926 in a tree  among them 7 primates:  60712 Chlorocebus tantalus  60710 Chlorocebus pygerythrus  54602 Macaca thibetana  9546 Macaca sylvanus  9544 Macaca mulatta  9541 Macaca fascicularis  9534 Chlorocebus aethiops | 68 | 13 |
| ND6 | 14439G>A | **P79S** | 283373 - Cypselurus hiraii (fish)  143332 - Exocoetus volitans (fish) | 2 | 1 |
|  | 14453G>A | **A74V** | 8387 - Bufo japonicus (toad)  30331 - Bufo gargarizans (toad)  8469 - Chelonia mydas (turtle)  34903 - Trachemys scripta (turtle)  8479 - Chrysemys picta (turtle)  262110 - Ursus thibetanus mupinensis (carnivore) | 6 | 3 |

Supplementary table S4. CPD in MITOMAP top-19 LHON variants. Amino acids that had been considered in previous sections (as they also cause other diseases) are colored grey.

| ***gene*** | ***mutation*** | ***amino acid change*** | ***species* with *CPD* *(NCBI IDs)*** | ***number of species with CPD*** | ***number of substitutions to pathogenic AA on a tree*** |
| --- | --- | --- | --- | --- | --- |
| ***top-3 mutations*** | | | | | |
| **ND4** | m.11778G>A ND4 | **R340H** | 0 | 0 | 0 |
| **ND1** | m.3460G>A ND1 | **A52T** | 0 | 0 | 0 |
| **ND6** | m.14484T>C ND6 | **M64V** | 0 | 0 | 0 |
| ***other mutations*** | | | | | |
| **ND1** | m.3376G>A | **E24K** |  |  |  |
|  | m.3635G>A | **S110N** | 0 | 0 | 0 |
|  | m.3697G>A | **G131S** |  |  |  |
|  | m.3700G>A | **A132T** | 9602 - Pongo pygmaeus pygmaeus (primate)  170189 - Alepocephalus tenebrosus (fish)  113382 - Onychodactylus fischeri (salamander)  280588 - Onychiurus orientalis (springtail) | 4 | 4 |
|  | m.3733G>A | **E143K** | 308094 - Hemieleotris latifasciata (fish)  308090 - Erotelis armiger (fish) | 2 | 1 |
|  | m.4171C>A | **L289M** | 696014 - Hylodes nasus (toad)  456503 - Thoropa taophora (frog)  357290 - Vaillantella maassi (fish)  109271 - Osteoglossum bicirrhosum (fish)  19 eels (1 substitution)  32411 - Perga condei (hymenopteran) | 24 | 6 |
| **ND3** | m.10197G>A | **A47T** |  |  |  |
| **ND4L** | m.10663T>C | **V65A** | 630 species, the closest to human are reptiles | 630 | 16 |
| **ND5** | m.13051G>A | **G239S** | 0 | 0 | 0 |
|  | m.13094T>C | **V253A** |  |  |  |
| **ND6** | m.14459G>A | **A72V** |  |  |  |
|  | m.14482C>A | **M64I** | 54971 - Arenaria interpres (bird) | 1 | 1 |
|  | m.14482C>G | **M64I** | the same | the same | the same |
|  | m.14495A>G | **L60S** | 103832 - Anguilla bengalensis labiate (fish) | 1 | 1 |
|  | m.14502T>C | **I58V** | 237 species, among them 2 are primates:  9600 - Pongo pygmaeus 756882 -Lepilemur hubbardorum | 237 | 34 |
|  | m.14568C>T | **G36S** | 124 species, closest to human are reptiles | 124 | 15 |
